# Clinical predictors of survival in malignant peripheral nerve sheath tumors of the head and neck: A cox regression and nomogram study

**DOI:** 10.1016/j.bjorl.2025.101702

**Published:** 2025-08-22

**Authors:** Sun LiNa, Tu Jianfei, Tian Zhifeng, Wang Xinbin, Wu Hui

**Affiliations:** a Department of Neurology, Lishui Central Hospital and Fifth Affiliated Hospital of Wenzhou Medical College, Zhejiang, China; b Department of Oncology, Lishui Central Hospital and Fifth Affiliated Hospital of Wenzhou Medical College, Zhejiang, China; c Department of Oral and Maxillofacial Surgery, Lishui Central Hospital and Fifth Affiliated Hospital of Wenzhou Medical College, Zhejiang, China

**Keywords:** Oral cancer, Head and neck cancer, Malignant Peripheral Nerve Tumors (MPNST), Survival, SEER database, COSMIC

## Abstract

**Objectives:**

Malignant Peripheral Nerve Sheath Tumors (MPNST) are rapidly progressing Schwann cell neoplasms. This study aimed to develop a practical clinical nomogram that predicts prognosis in patients with Head and Neck MPNST (HN-MPNST) using the Surveillance, Epidemiology, and End Results (SEER) database.

**Methods:**

We extracted clinical data from patients with HN MPNST between 2000 and 2020 from the SEER database. Patients were randomly divided into training cohort and validation cohorts. To estimate the chance of survival in patients with HN MPNST, we developed a nomogram. The nomogram performance was evaluated by discrimination and calibration. In addition, a decision curve analysis was conducted to evaluate the clinical usefulness of this newly developed model. Molecular data highlighting the most frequent mutations in MPNST were obtained from the Catalogue Of Somatic Mutations In Cancer (COSMIC) database.

**Results:**

In the primary cohort, 431 patients met the inclusion criteria to be entered into this study. The median Overall Survival (OS) was 3.9-years (95% CI 2.8–6.7), and the 1-, 3-, and 5-year OS rates were 75.2%, 52.6%, and 47.1%, respectively. We included 129 consecutive patients in the validation cohort. AJCC (American Joint Committee on Cancer) staging, Collaborative Stage (CS) extension, and CS tumor size were included in the nomogram. The calibration plots showed an agreement between the predictions and observations. Based on the clinical decision curve, the model had a net clinical benefit for patients with MPNST. Analysis of COSMIC data revealed frequent mutations of MPNST in NF1 (22%), SMARCB1 (21%), TP53 (12%), SUZ12 (19%), and less commonly in BRAF and CDKN2A (each 2%).

**Conclusion:**

Radiotherapy improves survival in patients with metastatic disease or tumors ≥ 6 cm. Using this nomogram can assist in clinical decision making, as it has satisfactory accuracy. However, an additional external validation is required.

**Level of evidence:**

2.

## Highlights

• Radiotherapy improves survival in cases with metastatic disease.
• Surgery and radiotherapy are important in patients with tumors ≥6 cm.
• Chemotherapy has limited efficacy in reducing the mortality of MPNST.
• An effective survival prediction model for patients with head and neck MPNST.

## Introduction

Malignant Peripheral Nerve Sheath Tumors (MPNST) are rare and aggressive malignancies originating from peripheral nerves or nerve sheaths, accounting for 5%‒10% of soft tissue sarcomas. Although they can occur at any site in the body, MPNST of the head and neck are relatively rare, accounting for only 2%‒9% of all MPNST.^1^,^2^ Due to their rarity and anatomical location, MPNST of the head and neck present unique clinical challenges in terms of diagnosis, management, and prognosis.

These tumors may arise sporadically or in the setting of Neurofibromatosis type 1 (NF1), a genetic disorder characterized by the development of multiple benign neurofibromas that may undergo malignant transformation into MPNST.^3^^,^^4^ Almost half of all cases are caused by defects in the neurofibromatosis type 1 gene located on chromosome 17; fewer than 10% of cases are radiation-induced (post-radiation sarcomas); and the rest are sporadic cases of unknown etiology.^5^ Owing to the complex anatomy of this region, the involvement of critical structures and vital organs often complicates surgical resection and may limit the efficacy of adjuvant therapies.^6^ Consequently, MPNSTs in the head and neck are associated with poor clinical outcomes, with reported 5-year survival rates ranging from 34% to 64%.^3^,^5^

Identification of clinical predictors of survival in patients with MPNST of the head and neck is crucial for guiding treatment decisions and improving patient outcomes. Previous studies have suggested that specific demographic and tumor-related factors may influence prognosis in this patient population. For instance, advanced age at diagnosis, non-Caucasian race, and larger tumor size have been associated with worse survival outcomes. In addition, the impact of treatment modalities such as radiotherapy and chemotherapy on survival remains a topic of interest and controversy.^1^^,^^6^

Despite current findings, further research is needed on prognostic factors for MPNSTs of the head and neck. We aimed to create a predictive model using Cox regression and nomogram analysis to help clinicians estimate survival likelihood and optimize treatment strategies for these patients.

## Methods

### Patient data

A population-based search for patients diagnosed with MPNST of the head and neck was performed using the case-listing session protocol of the National Cancer Institute’s SEER database (www.seer.cancer.gov); the database is a widely used cancer registry that covers an estimated 50% of the US population. The SEER database has been validated independently for analysis of both head and neck malignancies and MPNST.^7^, ^8^, ^9^ Access to the SEER database does not need formal ethics approval and is covered by its open access policy. No internal review board approval was required in this study because the database uses publicly available information with no personal identifiers.

For patients diagnosed with MPNST from 2000 to 2020, the widest date ranges available in the latest version of the SEER software, were reviewed. Histologic ICD-0−3 codes were used to include malignant peripheral nerve sheath tumor (9540/3) and malignant neurilemmoma (9560/3) in the query. Site-specific codes were used to confirm that the tumor originated in the head and neck.^10^ The following primary data were extracted from the database for analysis: age at diagnosis, sex, race, histologic subtype (ICD), primary site of the tumor, tumor extent and tumor size from both Extent of Disease (EOD) and Collaborative Stage (CS) coding methods, tumor stage, chemotherapy status, treatment with surgery and/or radiation therapy, cause of death, vital status, and survival months. Where available, TNM staging was recorded as explicitly listed in the SEER registries for all patients diagnosed between 2000 and 2020. For cases diagnosed prior to 2003, TNM stage was retrospectively determined, where possible without ambiguity, using CS and EOD staging codes for tumor size, extent, lymph node involvement, and evidence of distant metastasis using the classification criteria determined by the American Joint Committee on Cancer (AJCC). TNM staging and grade classification were then used to determine the stage at presentation (I‒IV).

Inclusion Criteria were as follows: 1) The patients were diagnosed with malignant peripheral nerve sheath tumors of the head and neck. 2) The years of diagnosis ranging from 2000 to 2020. 3) Patients with available clinical data for the predictive factors studied, including age, sex, race, stage, receipt of radiation therapy, receipt of chemotherapy, primary site of the tumor, extension, tumor size and vital status, and survival months. Exclusion Criteria: 1) Patients with a history of other cancer; 2) No evidence of primary tumor; and 3) Patients with unknown information of primary site, chemotherapy, survival months, or other important demographic, clinical, pathologic, and treatment variables. Patients with MPNSTs who met the criteria were randomly assigned to the training cohorts and validation cohorts.

### Data collection

The following demographic and clinical data were obtained: Age, Sex, Race, extension, tumor size, AJCC Stage, primary site, Chemotherapy status, and Surg/Rad status. The data of outcome and Clinical Predictors of Survival were also collected.Among 560 initially identified cases, 129 (23.0%) were excluded due to missing values in key variables such as tumor size or AJCC stage, potentially introducing selection bias.

### Statistical analysis

Descriptive statistics were calculated for all variables. OS curves were generated using Kaplan-Meier method and survival differences were tested with log-rank test. Univariate and multivariate Cox proportional hazards regression models were used to assess predictive performance. The dataset was randomly divided into 7:3 training and validation cohorts. Non-normal data were presented as median (interquartile ranges). Categorical variables were analyzed using Chi-Square or Fisher's exact test, while continuous variables were analyzed using Student’s *t*-test or rank-sum test. Cox regression analysis in the training cohort screened independent risk factors and built a prediction nomogram for Clinical Predictors of Survival. Nomogram performance was assessed using ROC and calibration curve. Decision curve analysis determined net benefit threshold. Statistical analyses were conducted using R software (version 4.2.2) and SPSS23 software (IBM Corp., Armonk, NY, USA).

## Results

### Patient characteristics

**Fig. 1:**
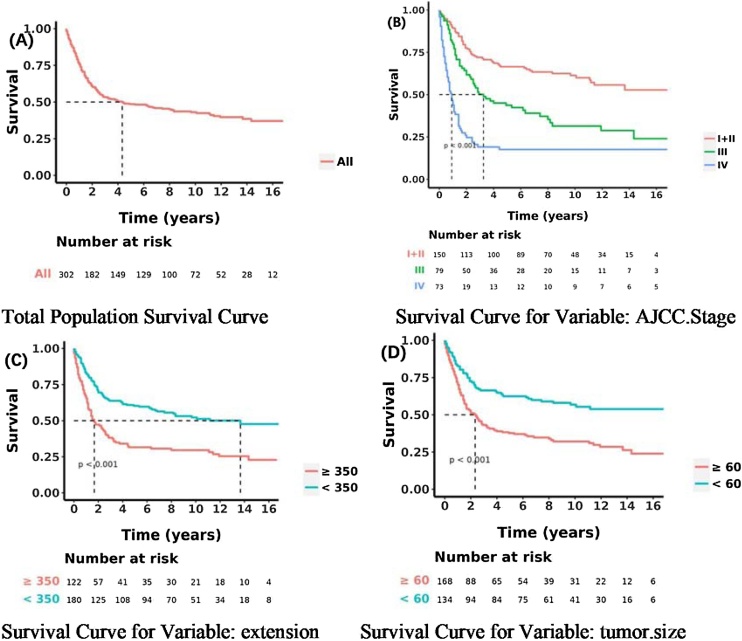
OS for the MPNST patients using Kaplan-Meier analysis and log-rank test. (A) Total Population Survival Curve. (B) OS among MPNST patients based on I‒II stage, III stage, and IV stage. (C) OS among MPNST patients based on extension. (D) OS among MPNST patients based on tumor size.

Fig. 1 illustrates data assembly process. The baseline demographic and clinical characteristics of the study cohorts were analyzed to provide an overview of the patient population (Table 1). In the training cohort (*n* = 302) and internal test cohort (*n* = 129), the distribution of patients across age groups did not show statistically significant differences (*p* = 0.746). For patients aged 45-years or older, the proportions were similar in the training cohort (56.0%) and the internal test cohort (54.3%). Additionally, the racial distribution differed significantly between the two cohorts (*p* = 0.045), with a higher percentage of white patients in the internal test cohort (84.5%) compared to the training cohort (76.5%). The sex distribution did not show significant differences between the training and internal test cohorts (*p* = 0.546). There were higher proportions of female patients in both cohorts, with 47.4% in the training cohort and 44.2% in the internal test cohort.

**Table 1:** Patient demographics and baseline characteristics.

Characteristic	Cohort		p-Value^b^
	Training cohort (*n* = 302)^a^	Internal test cohort (*n* = 129)^a^	
Age			0.746
≥45	169 (56.0%)	70 (54.3%)	
<45	133 (44.0%)	59 (45.7%)	
Race			0.055
White	231 (76.5%)	109 (84.5%)	
Black	37 (12.3%)	15 (11.6%)	
Other	34 (11.3%)	5 (3.9%)	
Sex			0.546
Female	143 (47.4%)	57 (44.2%)	
Male	159 (52.6%)	72 (55.8%)	
Extension			0.304
≥350	122 (40.4%)	59 (45.7%)	
<350	180 (59.6%)	70 (54.3%)	
Tumor size			0.682
≥60	168 (55.6%)	69 (53.5%)	
<60	134 (44.4%)	60 (46.5%)	
AJCC Stage			0.721
I + II	151 (50.0%)	62 (48.1%)	
III	80 (26.5%)	39 (30.2%)	
IV	71 (23.5%)	28 (21.7%)	
Primary site			0.985
Soft Tissue	232 (76.8%)	98 (76.0%)	
Cranial Nerves Other Nervous System	46 (15.2%)	21 (16.3%)	
Brain	11 (3.6%)	5 (3.9%)	
Other	13 (4.3%)	5 (3.9%)	
Chemotherapy status			0.423
No/Unknown	228 (75.5%)	102 (79.1%)	
Yes	74 (24.5%)	27 (20.9%)	
Surg/Rad status			0.337
Surgery	172 (57.0%)	67 (51.9%)	
Rad + Sur	130 (43.0%)	62 (48.1%)	

a n (%).

b Pearson’s Chi-Squared test; Fisher’s exact test.

When examining clinical characteristics, such as extension, tumor size, AJCC stage, primary site, chemotherapy status, and Surg/Rad status, no statistically significant differences were observed between the training and internal test cohorts. In terms of extension, the distribution of patients with extension ≥ 350 mm and < 350 mm was similar between the two cohorts. Furthermore, the proportion of patients with a tumor size ≥ 60 mm and < 60 mm did not differ significantly between the training and internal test cohorts. The distribution of AJCC stage I + II, III, and IV was also comparable between the two cohorts. Moreover, the primary site of the tumor and the status of chemotherapy and surgery/radiation treatment did not show significant variations between the training and internal test cohorts.

### Univariate and multivariate cox regression analysis

Univariate analyses were used to compare the indices between different outcome groups in the following Table 2. The results of the univariate and multivariate Cox regression analyses for OS are shown in Table 2, Table 3. Kaplan-Meier survival curves were plotted for the three categorical variables of extension, tumor size, and AJCC stage. Differences in the MPNST survival time distributions were examined using the log-rank method. The differences in the overall survival time distributions between the groups for these variables were statistically significant (*p* < 0.05). Variables in the univariate Cox regression that were statistically significant (*p* < 0.05) were incorporated into the multivariate analysis. In the multivariate analysis of OS, variables including sex, extension, tumor size, and AJCC stage were all statistically significant. According to multivariate analysis, the outcomes were improved in patients with lower extension, lower tumor size, and lower stage.

**Table 2:** Results of univariate Cox regression.

Characteristic	*n*	Event (*n*)	HR	95% CI	p-value
Age (year)					
≥45	169	91	‒	‒	
<45	133	82	1.25	0.93, 1.69	0.139
Race					
White	231	135	‒	‒	
Black	37	22	1.04	0.67, 1.64	0.851
Other (American Indian/AK Native, Asian/Pacific Islander)	34	16	0.72	0.43, 1.20	0.207
Sex					
Female	143	74	‒	‒	
Male	159	99	1.39	1.03, 1.89	0.031
Extension (mm)					
≥350	122	89	‒	‒	
<350	180	84	0.48	0.35, 0.64	<0.001
tumor size (mm)					
≥60	168	116	‒	‒	
<60	134	57	0.48	0.35, 0.66	<0.001
AJCC Stage					
I + II	151	60	‒	‒	
III	80	54	2.10	1.45, 3.03	<0.001
IV	71	59	4.77	3.31, 6.88	<0.001
Primary site					
Soft Tissue	232	143	‒	‒	
Cranial Nerves Other Nervous System	46	21	0.65	0.41, 1.02	0.061
Brain	11	3	0.33	0.11, 1.05	0.060
Other	13	6	0.65	0.29, 1.48	0.305
Chemotherapy status					
No/Unknown	228	117	‒	‒	
Yes	74	56	1.88	1.37, 2.60	<0.001
Surg/Rad status					
Surgery	172	99	‒	‒	
Rad + Sur	130	74	0.86	0.63, 1.16	0.316

HR, Hazard Ratio; CI, Confidence Interval.

**Table 3:** Results of multivariate Cox regression for training cohort.

Characteristic	*n*	Event (*n*)	HR	95% CI	p-value
Sex					
Female	143	74	‒	‒	
Male	159	99	1.37	1.00, 1.88	0.050
Extension (mm)					
≥350	122	89	‒	‒	
<350	180	84	0.53	0.39, 0.73	<0.001
Tumor size (mm)					
≥60	168	116	‒	‒	
<60	134	57	0.57	0.39, 0.83	0.003
AJCC Stage					<0.001
I + II	150	60	‒	‒	
III	79	53	1.24	0.80, 1.93	0.343
IV	73	60	3.28	2.17, 4.95	<0.001
Chemotherapy status					
No/Unknown	228	117	‒	‒	
Yes	74	56	0.95	0.66, 1.36	0.776

HR, Hazard Ratio; CI, Confidence Interval.

### Predictive model

The candidate predictors, Age, Race, Sex, extension, tumor size, AJCC Stage, primary site, chemotherapy status, and Surg/Rad status, were included in the original model, which were then reduced to 5 potential predictors using univariate analysis performed in the training cohort. Further multivariate Cox analyses were performed on the training cohorts. Results are shown in the following Table 3. The results of the final Multivariate Cox regression analysis of the Training Cohort are shown in Table 4.

**Table 4:** Results of final multivariate Cox regression for training cohort.

Characteristic	*n*	Event (*n*)	HR	95% CI	p-value
Extension					
≥350	122	89	‒	‒	
<350	180	84	0.55	0.41, 0.75	<0.001
Tumor size					
≥60	168	116	‒	‒	
<60	134	57	0.62	0.43, 0.89	0.010
AJCC Stage					<0.001
I + II	150	60	‒	‒	
III	79	53	1.36	0.89, 2.08	0.158
IV	73	60	3.48	2.37, 5.11	<0.001

HR, Hazard Ratio; CI, Confidence Interval.

### Construction and validation of prognostic nomogram

**Fig. 2:**
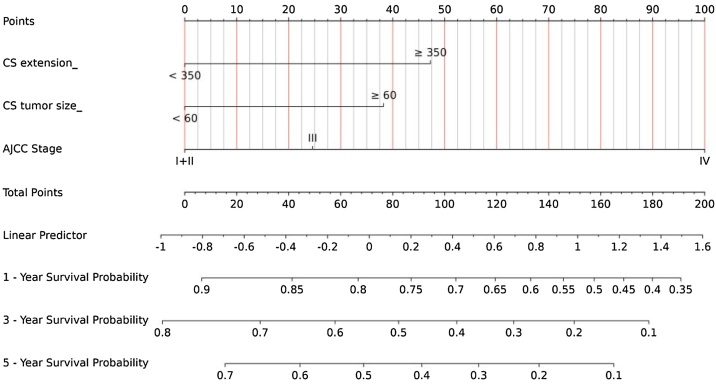
Nomogram predicting 1-, 3-, and 5-year OS for patients with MPNST.

**Fig. 3:**
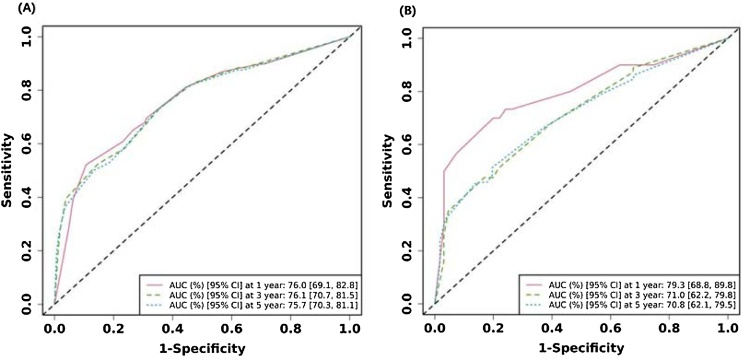
Receiver operating characteristic curve analysis for evaluating the accuracy of the 1-, 3-, and 5-year nomogram. (A) Training group and (B) Validation group.

**Fig. 4:**
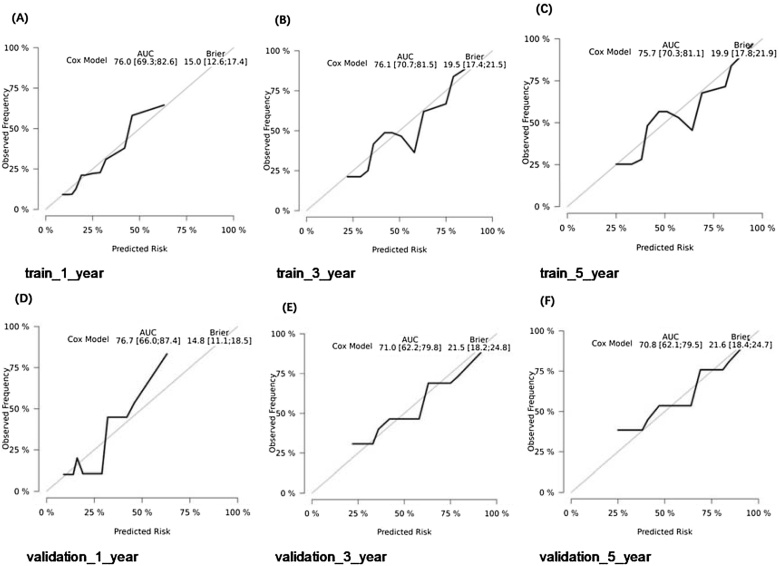
Calibration plots of the nomogram for predicting 1-, 3-, and 5-year OS. Nomogram-predicted OS is plotted on the x-axis; actual OS is plotted on the y-axis. (A–C) Training group and (D–F) Validation group.

**Fig. 5:**
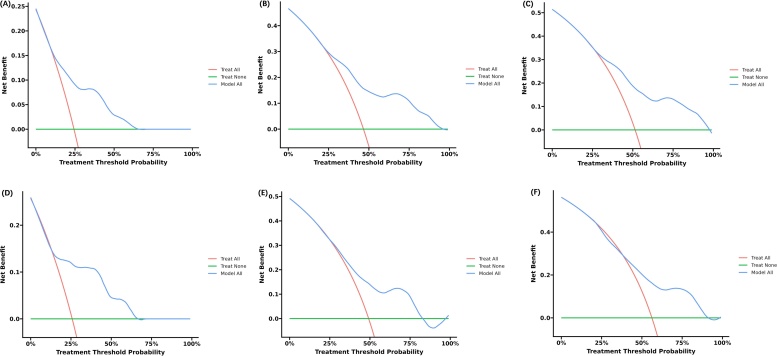
Decision curve analysis for evaluating the net benefit of nomogram and 6th AJCC TNM grading system. (A) 1-year net benefit in training cohort; (B) 3-year net benefit in training cohort; (C) 5-year net benefit in training cohort; (D) 1-year net benefit in validation cohort; (E) 3-year net benefit in validation cohort. (F) 5-year net benefit in validation cohort.

The final Cox model included three independent predictors (extension, tumor size, and AJCC Stage) and was developed as a simple-to-use nomogram, which is illustrated in the following figure (Fig. 2). ROC plots were generated using the “survivalROC” package for the training and validation group at different times, with the death rate expressed as a continuous variable. AUC was calculated for each plot (Fig. 3). In the training set, the prognostic model accurately predicted 1-, 3-, and 5-year mortality with AUCs of 0.760, 0.761, and 0.757, respectively. In the validation set, AUC values at 1-, 3-, and 5-years were 0.793, 0.710, and 0.708, respectively. These results indicate that this model is accurate. Fig. 4 shows the calibration plots for the nomogram. The DCA results indicate the model provides good net benefits to the patients with MPNST (Fig. 5). The survival difference between chemotherapy and no chemotherapy (*p* < 0.001) achieved statistical significance for OS, but doesn’t for DSS (*p* = 0.362). The survival difference between bimodal therapy and surgery, and different radiotherapy sequences did not achieve statistical significance for both OS and DSS, respectively (Table 5).

**Table 5:** Head-to-head Kaplan-Meier analysis of treatment modality.

Characteristic	Overall survival (OS)		Disease-specific survival (DSS)	
	Hazard ratio	p-Value	Hazard ratio	p-value
Chemotherapy vs. no chemotherapy	1.83 (1.40–2.39)	*p* < 0.001	0.85 (0.60–1.20)	*p* = 0.362
Surgery + radiation vs. surgery only	0.89 (0.69–1.13)	*p* = 0.337	0.87 (0.68–1.12)	*p* = 0.289
Surgery + radiation after surgery vs. surgery only	0.88 (0.68–1.14)	*p* = 0.347	0.84 (0.65–1.10)	*p* = 0.214
Surgery + radiation prior to surgery vs. surgery only	0.90 (0.54–1.51)	*p* = 0.694	1.09 (0.63–1.89)	*p* = 0.758

The AUCs of the model in different cohorts are shown in the following figures.

The calibration plots of the nomogram in the different cohorts are plotted in the following figures, which demonstrate a good correlation between the observed and predicted risks. The results showed that the original nomogram was still valid for use in the validation sets, and the calibration curve of this model was relatively close to the ideal curve, indicating that the predicted results were consistent with the actual findings.

### Decision curve analysis

The following figure displays the DCA curves related to the nomogram. A high-risk threshold probability indicates the chance of significant discrepancies in the model’s prediction when clinicians encounter major flaws while utilizing the nomogram for diagnostic and decision-making purposes. This study shows that the nomogram offers substantial net benefits for clinical application through its DCA curve.

### Survival analyses

**Fig. 6:**
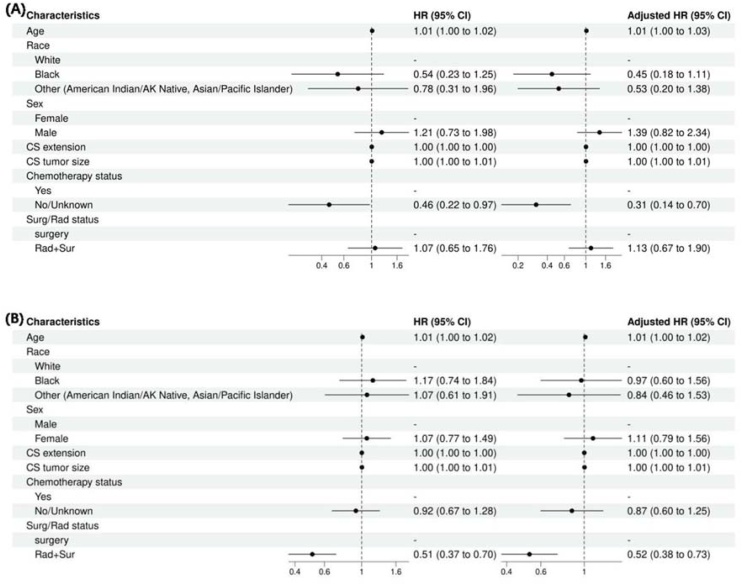
Forest plot analysis for stage I–II group (A) and stage III–IV group (B).

Kaplan–Meier survival curves were generated for the three categorical variables: cs extension, cs tumor size, and AJCC stage. Survival time differences for HN-MPNST were assessed using the log-rank test (Fig. 6), revealing statistically significant differences in overall survival distributions (p < 0.05). We conducted an analysis of OS for HN-MPNST patients utilizing Kaplan-Meier analysis and the log-rank test. (A) Overall Survival Curve for the Total Population. (B) OS among HN-MPNST patients categorized by I-II stage, III stage, and IV stage. (C) OS among MPNST patients stratified by CS extension. (D) OS among HN-MPNST patients differentiated by CS tumor size.

### Subgroup analyses

**Fig. 7:**
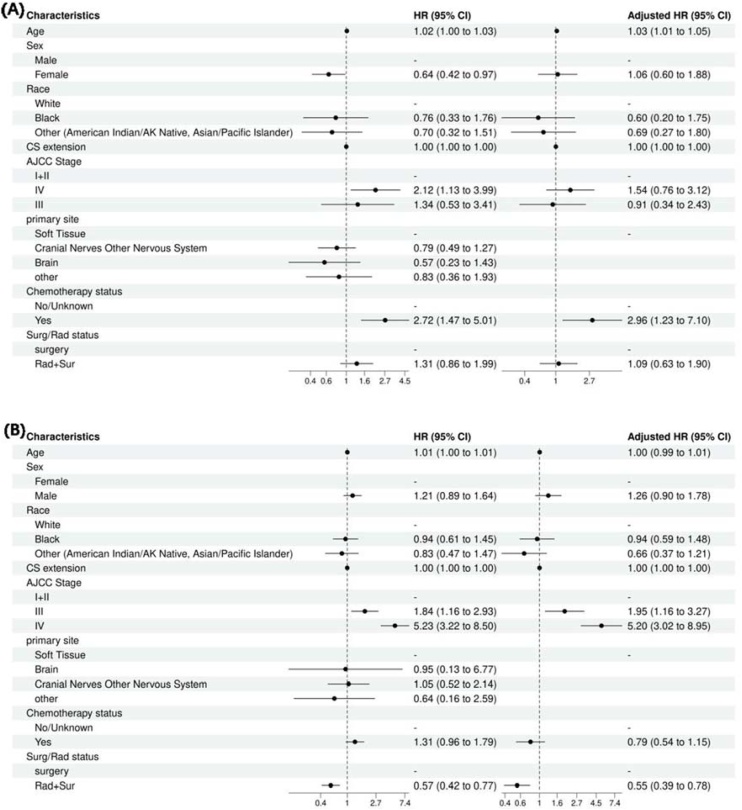
Forest plot for tumor size < 60 mm group (A) and tumor size ≥ 60 mm group (B). **Genetic Mutations in MPNST** The genetic mutations data for MPNST were extracted from COSMIC (https://cancer.sanger.ac.uk/cosmic, accessed on 01 February 2025) version GRCh38 COSMIC v101. A totalof 15,513 cases of soft tissue were evaluated for genetic mutations in the database. Inthe sub-selection category, nerve sheath was selected for data extraction (734/3515). In histology selection, Malignant peripheral nerve sheath tumour was selected, which was 113 cases (113/1263). In the sub-histology selection for MPNST, a total of 113 cases for which genetic analysis was conducted for MPNST were identified. The top 20 genes found mutated in MPNST were TP53 12% (samples tested = 287), NF1 22% (133), SMARCB1 21% (34), BRAF (2 %) (239), CDKN2A (2 %)(132), SUZ12 (19 %)(16), KRAS (2 %)(121), PDGFRA (2 %)(84), PIK3CA (1 %)(108), NRAS (2 %)(56), CTNNB1 (2 %)(54), H3F3B (3 %)(39), NF2 (3 %)(34), KMT2D (3 %)(32), RET (4 %)(24), PTCH1 (5 %)(19), BRCA1 (6 %)(18), PAX5 (6 %)(18), STAG2 (6 %)(18), CBL (6 %)(18). The top genes found mutated in MPNST (sub-histology selection is Epithelioid) were SMARCB1 (44 %)(16), NF1 (13 %)(16), CDKN2A (6 %)(16), TP53 (6 %)(16), KMT2D (6 %)(16), NF2 (6 %)(16).

The multivariate analysis model was next used to ascertain the independent effects of these variables on survival in patients with stage I/II and stage III/IV tumors as separate cohorts. For patients presenting at stage I/II tumors, age 1.01 (95% CI 1.00–1.01, *p* = 0.047) and chemotherapy status (no chemotherapy) 0.31 (95% CI 0.14‒0.70, *p* = 0.005) were independent determinants of OS (Fig. 6A). In patients with stage III/IV tumors, age 1.01 (95% CI 1.00–1.02, *p* = 0.007) and Surg/Rad status 0.52 (95% CI 0.38‒0.73, *p* < 0.001) (Fig. 6B). The model was also used to determine prognostic indicators for tumors 6 cm and > 6 cm as independent cohorts. For tumors size < 6 cm, age 1.03 (95% CI 1.01–1.05, *p* < 0.001) and chemotherapy status (YES) 2.96 (95% CI 1.23–7.10, *p* = 0.015) were independent determinants of OS (Fig. 7A). For tumors ≥ 6 cm, stage III at presentation 1.95 (95% CI 1.16–3.27, *p* = 0.011), stage IV at presentation 5.20 (95% CI 3.02–8.95, *p* < 0.001), and Surg/Rad status 0.55 (95% CI 0.39‒0.78, *p* < 0.001) were independent predictors of OS (Fig. 7B).

## Discussion

In the current study, we developed and validated a nomogram for predicting the prognosis of Malignant Peripheral Nerve Sheath Tumors (MPNSH) of the Head and Neck, based on a cohort of patients. The main predictors incorporated into the nomogram included tumor extension, tumor size, and stage, which were statistically significant in the multivariate Cox regression analysis (Fig. 2). Nomograms rely on easy-to-use digital interfaces, improved accuracy, and clearer prognoses to help doctors make better decisions.

Using the SEER database, we created a prognostic model to determine the prognosis of HD MPNST patients based on large-sample data. A total of 431 patients were enrolled in this study. MPNSTs exhibit resistance to the majority of systemic therapies, leading to a diminished survival rate in advanced settings.^11^ Our investigation revealed that chemotherapy serves as a negative prognostic factor for OS but does not exhibit a significant association with DSS. This observation could be attributed to its limited efficacy and adverse impact on the quality of life for patients. Conventional chemotherapy appears to have limited efficacy in reducing the mortality of MPNST, prompting questions regarding its role in treatment.^12^ Some studies have indicated the ineffectiveness of adjuvant chemotherapy for MPNST treatment, with the exception of epirubicin and ifosfamide. These two agents have been shown to enhance the median survival time, increasing it from 45 to 75-months when used in conjunction with local therapies such as amputation, wide resection followed by radiation, or pre-operative radiation followed by surgery.^13^ Perioperative chemotherapy has demonstrated limited effectiveness in improving disease-specific overall survival in patients with localized malignant peripheral nerve sheath tumors,^12^^,^^14^ and its toxicity must be considered. Although adjuvant chemotherapy is widely employed in the treatment of MPNST, its efficacy is a subject of debate.^15^

This study supports previous research showing that radiotherapy improves the survival of patients with metastatic disease. In addition surgery and radiotherapy are prognostically important in patients with larger tumors.^1^ This suggests a control effect of radiotherapy in patients with metastatic lesions and a large tumor burden. High-grade lesions or tumors larger than 5 cm often warrant the recommendation of radiation therapy.^16^

The application of radiation therapy yields excellent local control as a long-term outcome.^17^ However, adjuvant radiation therapy does not confer survival benefits in MPNST. Some studies have explored its use to reduce tumor size and facilitate subsequent surgery.^18^ Brachytherapy and intraoperative electron radiation therapy have also been used for the treatment of MPNST. In a study by Wong et al., five-year local control rates were reported as 88% with brachytherapy and 51% with external beams.^19^ Achieving a cumulative dose of ≥60 Gy was essential for effective local disease control.^19^ The combination of brachytherapy and external beam radiation may enhance the efficacy.^20^

Our findings align with the existing literature in many respects. For instance, tumors size > 5 cm has been previously identified as a significant predictor, and surgery and radiotherapy are prognostically important in patients with tumors > 5 cm.^1^ In this study, a tumor size ≥ 6 cm was significant in both univariate and multivariate regression analyses and was an independent risk factor. Our cutoff value was adjusted to 6 cm, possibly due to differences in research data, and it is also consistent with the values reported in previous studies.^21^ In the prediction model, we observed that when the cutoff value for tumor size was set to 5 cm or 6 cm, a statistical difference emerged in the Cox regression analysis. However, a 6 cm cutoff value demonstrated a superior predictive value.

However, our study uniquely highlights the importance of tumor extension, which has not been emphasized in previous studies. This discrepancy could be attributed to differences in the cohort characteristics or methodologies. The challenges in patient treatment are frequently compounded by local progression and distant metastasis.^22^^,^^23^ As Magnetic Resonance Imaging (MRI) technology continues to advance, its sensitivity and specificity for tumor diagnosis are consistently improving. Broski et al. demonstrated that perilesional edema, cystic degeneration, or necrosis, along with irregular margins, can achieve 100% specificity when all three signs are present simultaneously.^21^,^24^

Multivariate analysis of disease-specific overall survival highlighted that deep-seated tumors were associated with adverse prognostic outcomes.^14^ In multivariate analysis, deep location was one of the poor prognostic factors for OS in patients with histologically confirmed MPNST.^4^ Tumor extension provides valuable information for both diagnosis and prognosis. Despite these capabilities, it is seldom employed as a gold standard in clinical practice.

Tumors located in the extremities exhibit a favorable OS compared to truncal locations, although this trend is not observed in Progression-Free Survival (PFS). Conversely, tumors in the head and neck show favorable PFS but not OS when compared to those in the trunk.^25^ In this analysis, we observed that the primary tumor site did not significantly affect the survival of patients with HN MPNST.

These tumors manifest with comparable frequency in males and females, with a certain series suggesting a male preponderance.^26^ Our current study revealed a notable male preponderance, exerting a significant impact on the overall survival of the disease (p = 0.05). However, drawing definitive conclusions regarding sex as a significant prognostic factor remains challenging owing to the limited size of our series and the possibility of referral bias. Therefore, we excluded this from the predictive model.

MPNST commonly manifests in individuals aged 30–50. Approximately 50% of MPNST cases are linked to NF1, whereas approximately 10% of MPNST patients have a history of radiation exposure. The presence of PN and ANNUBP has malignant potential for the development of MPNST. On comparing the OS between the HN MPNST and other head and neck soft tissue sarcomas with confirmed molecular diagnosis (Ewing sarcoma, Ewing-like sarcoma with CIC-rearrangements, rhabdomyosarcoma and synovial sarcoma),^27^, ^28^, ^29^ MPNST patients had the worst outcome. Our research indicates that the model effectively predicts the survival of patients with HN MPNST. The developed nomogram offers several clinical implications. Firstly, it provides a quantitative tool for clinicians to predict the MPNST of the Head and Neck prognosis more accurately than traditional methods, aiding in better risk stratification. Moreover, early identification of high-risk individuals using this nomogram can lead to timely interventions, potentially reducing morbidity and mortality.

The rapid evolution of sequencing methodologies has generated a vast and diverse array of multi-omics data from MPNST studies. These comprehensive datasets have facilitated the discovery of key driver mutations in genes such as NF1, CDKN2A, PRC2 complex members, and TP53, while simultaneously revealing multiple promising therapeutic avenues. These include combination therapies involving MEK inhibitors,^30^ along with novel agents targeting HDAC, SHP2, TYK2, and CDK4/6 pathways.^30^, ^31^, ^32^ Furthermore, emerging evidence from these datasets highlights the therapeutic potential of immunomodulatory approaches in MPNST treatment.^33^

Research indicates that soft tissue sarcomas typically exhibit a low tumor mutational burden (TMB), ranging between 1 and 2.5 mutations per megabase (mutations/Mb).^34^ Nevertheless, a comprehensive study involving 100,000 diverse cancer cases revealed that 8.2% of malignant peripheral nerve sheath tumors (MPNSTs) displayed a significantly higher mutation rate, exceeding 20 mutations/Mb.^35^ Supporting this finding, a separate investigation analyzing whole-exome sequencing data from 12 MPNST patient samples demonstrated a wide variation in somatic coding variants, with counts per tumor spanning from 7 to 472 and a median of 63 mutations.^36^

The role of molecular classification has grown substantially in both tumor diagnostics and therapeutic decision-making. This approach to subtype-specific categorization has facilitated the creation of personalized treatment protocols, leading to marked enhancements in patient outcomes.^37^ Parallel to these advancements, multiple research efforts have employed computational methods to delineate distinct gene expression profiles associated with MPNST subtypes. Notably, Holand and colleagues conducted clustering analyses using MPNST transcriptomic data, revealing two immune-associated subgroups with significant prognostic implications.^38^ Their findings suggested EGFR, EZH2, KIF11, PLK1, and RRM2 as promising molecular targets for MPNST therapy. The most extensive genomic investigation of MPNSTs conducted thus far was carried out by the GeM consortium, demonstrating that molecular expression profiles provide superior prognostic value compared to traditional clinical and histopathological assessments.

Our study has several limitations that should be acknowledged. Firstly, the stages were organized based on the 6th AJCC staging system, which could limit its effectiveness. Secondly, our nomogram was constructed based on the SEER database, in which partial patient information was lost. Thirdly, there may be potential unmeasured confounders that were not included in our model. Finally, this was a retrospective, cohort study. In the current situation, given that MPNST of the Head and Neck is a very rare soft tissue sarcoma, the most reliable way to study the prognosis of this disease is using public database. External validation in diverse populations is essential for confirming the generalizability of our findings.

## Conclusion

Using this nomogram can assist in clinical decision-making as it has satisfactory accuracy. However, an additional external validation is required. Future research should aim to validate our nomogram externally in different populations and settings. Additionally, integrating novel predictors or biomarkers could enhance the predictive accuracy of the nomogram, warranting further investigations.

## ORCID IDs

Sun Lina: 0000-0003-0845-3218, Tu Jianfei: 0000-0002-1708-893X, Tian Zhifeng: 0009-0005-4982-8073, Wang Xinbin: 0009-0002-6462-1124

## CRediT authorship contribution statement

Sun LiNa, WuHui: Conceptualization; methodology.

Sun LiNa: Data curation; writing-original draft preparation.

Tu Jianfei, Sun LiNa: Visualization; investigation.

Tu Jianfei: Supervision.

Tian Zhifeng, WXB: Software; validation.

WuHui: Writing-reviewing and editing.

Wang Xinbin: Funding acquisition.

All the authors contributed to the article and approved the submitted version, and wrote the manuscript.

## Ethics statement

No personal identifying information was used in this study. Hence, this study did not require Institutional Review Board approval or patient informed consent.

## Authorship clarified

All authors agreed with the content, all gave explicit consent to submit, and we obtained consent from the responsible authorities at the institution where the work was carried out, before the work was submitted.

## Funding

This study was supported by the Zhejiang Provincial Medical and Health Science and Technology Plan Project (2025KY1948) and the Public Welfare Technology Application Research Program of Lishui (2020SJZC046).

## Data availability statement

All data were downloaded from the SEER (https://seer.cancer.gov/). Data from this study are available to any interested researchers upon reasonable request from the corresponding author.

## Declaration of competing interest

The authors declare no conflicts of interest.
